# Production of Hydrogen-Rich Syngas via Biomass-Methane Co-Pyrolysis: Thermodynamic Analysis

**DOI:** 10.3390/polym17192695

**Published:** 2025-10-05

**Authors:** Haiyan Guo, Zhiling Wang, Kang Kang, Dongbing Li

**Affiliations:** 1 Key Comprehensive Laboratory of Forestry, College of Forestry, Northwest A&F University, Yangling 712100, China; guohaiyan9128@gmail.com; 2 College of Forestry, Shanxi Agricultural University, Taigu 030801, China; 3 Biorefining Research Institute (BRI) and Department of Chemical Engineering, Lakehead University, Thunder Bay, ON P7B 5E1, Canada; kkang3@lakeheadu.ca; 4 Nottingham Ningbo China Beacons of Excellence Research and Innovation Institute, University of Nottingham Ningbo China, Ningbo 315048, China

**Keywords:** biomass-methane co-pyrolysis, hydrogen-rich syngas, thermodynamic equilibrium analysis, Gibbs free energy minimization

## Abstract

This study presents a thermodynamic equilibrium analysis of hydrogen-rich syngas production via biomass–methane co-pyrolysis, employing the Gibbs free energy minimization method. A critical temperature threshold at 700 °C is identified, below which methanation and carbon deposition are thermodynamically favored, and above which cracking and reforming reactions dominate, enabling high-purity syngas generation. Methane addition shifts the reaction pathway towards increased reduction, significantly enhancing carbon and H_2_ yields while limiting CO and CO_2_ emissions. At 1200 °C and a 1:1 methane-to-biomass ratio, cellulose produces 50.84 mol C/kg, 119.69 mol H_2_/kg, and 30.65 mol CO/kg; lignin yields 78.16 mol C/kg, 117.69 mol H_2_/kg, and 19.14 mol CO/kg. The H_2_/CO ratio rises to 3.90 for cellulose and 6.15 for lignin, with energy contents reaching 43.16 MJ/kg and 52.91 MJ/kg, respectively. Notably, biomass enhances methane conversion from 25% to over 53% while sustaining a 67% H_2_ selectivity. These findings demonstrate that syngas composition and energy content can be precisely controlled via methane co-feeding ratio and temperature, offering a promising approach for sustainable, tunable syngas production.

## 1. Introduction

Global economic expansion and fossil fuel depletion, coupled with environmental challenges, are driving the transition to sustainable energy systems [1,2,3]. Biomass energy stands out as a carbon-neutral, widely available renewable resource, capable of conversion into solid, liquid, and gaseous fuels, making it a cornerstone of future energy infrastructure [4,5,6]. Among thermochemical technologies, gasification is especially attractive for producing syngas (a mixture of H_2_ and CO), an essential feedstock for advanced fuels and chemicals [7,8,9,10,11].

However, the low hydrogen-to-carbon (H/C) and high oxygen-to-carbon (O/C) ratios of biomass inherently limit syngas quality, resulting in low H_2_/CO ratios, reduced heating value, and substantial tar formation—all detrimental to downstream utilization [12,13]. Thus, upgrading biomass-derived syngas is vital. This study investigates methane co-processing—a hydrogen-rich co-reactant—to overcome these limitations and enable high-value, hydrogen-rich syngas production [2,12]. Previous studies demonstrated experimental feasibility (e.g., Palumbo et al. [13] showed efficient hydrogen-enriched syngas via high-temperature biomass–methane gasification with steam). Lalsare et al. [7] clarified methane’s mechanistic role, activating steam reforming and suppressing methane formation, thereby driving selective hydrogen production. Methane enrichment also enhances combustion performance in dual-fuel systems [1].

Despite experimental progress, most prior work focuses on specific conditions and outcomes, lacking fundamental thermodynamic insight, especially at the initial biomass–methane co-pyrolysis stage, where complex competing reactions occur. Thermodynamic equilibrium analysis based on Gibbs free energy minimization is ideal, but prior models often omit critical aspects like tar formation [6]. This work addresses these gaps by building an inclusive thermodynamic framework for biomass–methane co-pyrolysis, explicitly incorporating tar formation. Using cellulose and lignin as model biomass compounds, we systematically evaluate temperature and feedstock ratio effects, aiming to elucidate synergistic mechanisms and guide optimization for high-yield hydrogen production.

## 2. Materials and Methods

### 2.1. Materials

Pure cellulose (C_6_H_10_O_5_) and lignin (C_11_H_12_O_4_) are used as model biomass components [14,15], enabling controlled study of gasification chemistry without interference from heteroatoms (S, N) or minerals (K, Na). Thermodynamic properties for all species are sourced mainly from HSC Chemistry 6.0 (Table 1). A custom database was created for cellulose/lignin [15], while other species used default library values.

### 2.2. Related Reactions

The co-pyrolysis of biomass and methane is a complex process involving multiple simultaneous reactions [11,16]. The primary reaction is pyrolysis or gasification of methane and biomass (Equations (1)–(3)), which involves various competing side reactions [6,11], including the Boudouard reaction (Equation (4)), methanation (Equations (5)–(7)), the water–gas reaction (Equation (8)), the water–gas shift reaction (Equation (9)), and reforming reactions (Equations (10) and (11)). The specific reaction equations are as follows:

Pyrolysis or gasification:

(1) $${\mathrm{CH}}_{4}(g)⇌C(s)+2H_{2}(g)$$

(2) $$C_{6}H_{10}O_{5}(s)⇌C(s){+5H}_{2}(g)+5\mathrm{CO}(g)$$

(3) $$C_{11}H_{12}O_{4}(s)⇌7C(s)+6H_{2}(g)+4\mathrm{CO}(g)$$

Boudouard reaction:

(4) $$2\mathrm{CO}(g)⇌C(s)+{\mathrm{CO}}_{2}(g)$$

Methanation:

(5) $$C(s)+2H_{2}(g)⇌{\mathrm{CH}}_{4}(g)$$

(6) $$\mathrm{CO}(g)+3H_{2}(g)⇌{\mathrm{CH}}_{4}(g)+H_{2}O(g)$$

(7) $${\mathrm{CO}}_{2}(g)+4H_{2}(g)⇌{\mathrm{CH}}_{4}(g)+2H_{2}O(g)$$

Water–gas reaction:

(8) $$C(s)+H_{2}O(g)⇌\mathrm{CO}(g)+H_{2}(g)$$

Water–gas shift reaction (WGSR):

(9) $$\mathrm{CO}(g)+H_{2}O(g)⇌{\mathrm{CO}}_{2}(g)+H_{2}(g)$$

Reforming reaction

(10) $${\mathrm{CH}}_{4}(g)+H_{2}O(g)⇌\mathrm{CO}(g)+3H_{2}(g)$$

(11) $${\mathrm{CH}}_{4}(g)+{\mathrm{CO}}_{2}(g)⇌2\mathrm{CO}(g)+2H_{2}(g)$$

Gibbs free energy change (Δ*G*) is a fundamental concept in thermodynamics, used to determine the spontaneity and equilibrium state of chemical reactions or physical processes under given conditions. It is defined as [17]:

(12) ΔG=ΔH−TΔS

where Δ*G* is minimized at equilibrium, Δ*H* represents the change in the system’s enthalpy, *T* denotes temperature in Kelvin, and Δ*S* represents the change in the system’s entropy.

Using the Reaction Equations module within HSC Chemistry 6.0, the temperature-dependent Δ*G* were systematically calculated for all considered reactions across the range of 100–1200 °C.

### 2.3. Determination of Equilibrium Compositions

A thermodynamic equilibrium model based on Gibbs free energy minimization was established to investigate biomass–methane co-pyrolysis systems, utilizing the Equilibrium Compositions module in the HSC Chemistry 6.0 software. The model considered cellulose and lignin (kg basis) as biomass components along with methane (kg basis) as co-reactants, with the product spectrum encompassing C, H_2_O, CH_4_, H_2_, CO_2_, CO, and tar. To improve predictive accuracy, tar formation was explicitly accounted for, enabling a more comprehensive representation of gasification behavior and secondary reaction pathways. Benzene (C_6_H_6_) was usually employed as a representative tar model compound for gasification [1,18]. The thermodynamic framework consistently applied ideal gas law assumptions at standard pressure (1 atm), with all species properties sourced from established databases and phase equilibria explicitly considered in all calculations. The analysis systematically evaluated the effects of temperature (100–1200 °C) and biomass-to-methane mass ratios (1:0, 1:0.25 or 4, 1:0.5 or 2, 1:1 or 1, 0:1) on the composition of product gas and the tendency of tar formation, providing fundamental insights into the co-pyrolysis behavior of these feedstocks.

### 2.4. Determination of Energy Recovery Efficiency

Heating value serves as a critical parameter for evaluating the energy quality of fuels. A higher heating value indicates greater energy release during combustion. Furthermore, the energy conversion efficiency during fuel transformation into other energy forms is equally essential. Consequently, in gasification processes, the energy recovery efficiency (also termed cold gas efficiency, CGE) represents a key performance indicator for assessing the technology’s efficacy [6,13]. The energy recovery efficiency is defined as [6]:

(13) $$E_{\eta }=\frac{E_{\mathrm{out}}}{E_{\mathrm{in}}}\times 100\%$$

(14) $$E_{\mathrm{in}}=E_{\mathrm{biomass}}+E_{{\mathrm{CH}}_{4}}=HHV_{\mathrm{biomass}}\times n_{\mathrm{biomass}}+LHV_{{\mathrm{CH}}_{4}}\times n_{{\mathrm{CH}}_{4}}$$

(15) $$E_{\mathrm{out}}=E_{C}+E_{{\mathrm{CH}}_{4}}+E_{H_{2}}+E_{\mathrm{CO}}=LHV_{C}\times n_{C}+LHV_{{\mathrm{CH}}_{4}}\times n_{{\mathrm{CH}}_{4}}+LHV_{H_{2}}\times n_{H_{2}}+LHV_{\mathrm{CO}}\times n_{\mathrm{CO}}$$

where *E*_η_, *E*_in_, and *E*_out_ represent energy recovery efficiency, input energy, and output energy, respectively. *HHV*_biomass_ represents the high heating value of the biomass, and *LHV*_i_ represents the low heating value of each chemical species contained in syngas, that is defined in Table 1, kg/mol. *n*_i_ represents the mole fraction of each chemical species contained in syngas, mol.

## 3. Results

### 3.1. Gibbs Free Energy Variation in Key Reactions

**Figure 1:**
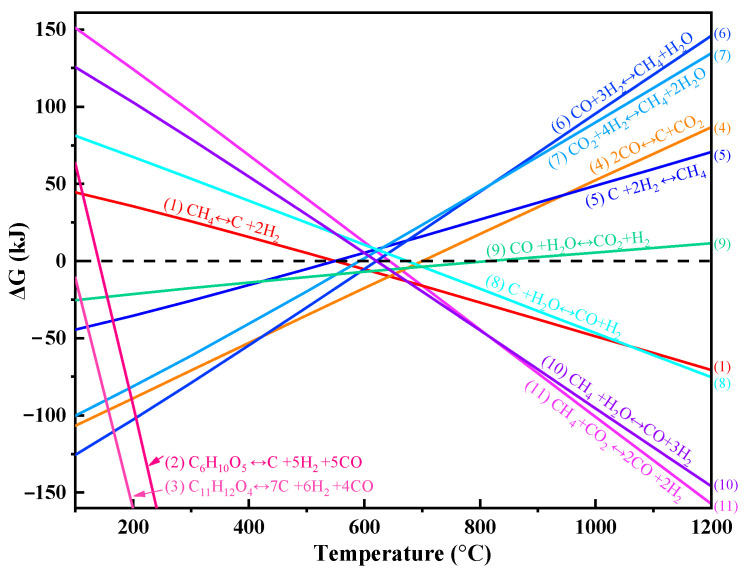
Variation in ΔG with temperature for various reactions during biomass-CH_4_ co-pyrolysis.

The Gibbs free energy changes (Δ*G*) of key biomass-methane co-pyrolysis reactions (Equations (1)–(11)) exhibit distinct temperature-dependent behavior between 100 and 1200 °C (Figure 1). The ΔG values for most reactions decreased with increasing temperature. The thermodynamic landscape demonstrates two characteristic regimes. When temperatures are below 700 °C, biomass-methane co-pyrolysis favors two competing pathways: methanation and carbon deposition [19]. Methanation reactions (Equations (5)–(7)), being exothermic and thermodynamically favorable (Δ*G* < 0), consume H_2_ to form CH_4_, while the Boudouard reaction (Equation (4)) promotes solid carbon deposition via CO disproportionation. Above 700 °C, methane cracking (Equation (1)) and reforming reactions (Equations (10) and (11)), become thermodynamically favorable, while the water-gas shift reaction (Equation (9)) approaches optimal equilibrium, maximizing H_2_ production. The combined effect of these reactions leads to enhanced syngas yields with improved H_2_/CO ratios, particularly under steam-rich conditions.

### 3.2. Equilibrium Product Distribution

#### 3.2.1. Pure Feedstock Pyrolysis

**Figure 2:**
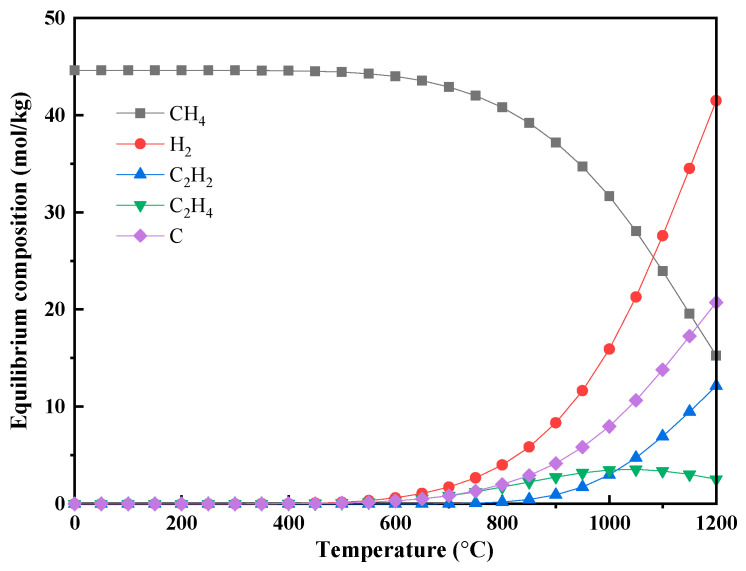
Equilibrium compositions of methane pyrolysis under different temperatures (*p* = 1 bar, 1 kg CH_4_).

**Figure 3:**
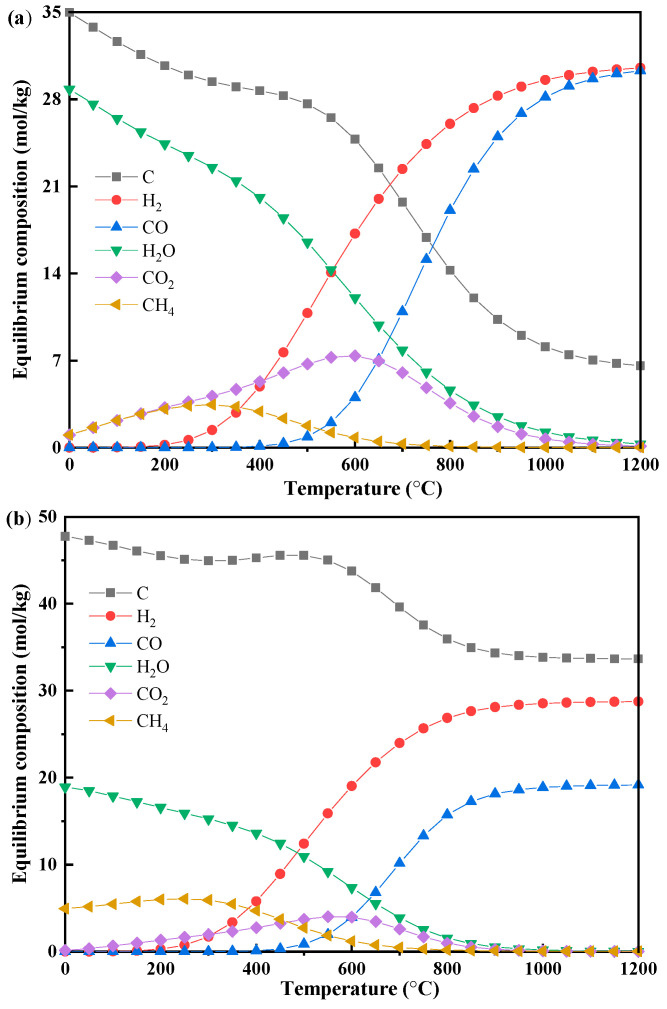
Equilibrium compositions of biomass gasification under different temperatures (*p* = 1 bar, biomass 1 kg): (**a**) CE; (**b**) LG.

The equilibrium compositions for pure methane, cellulose, and lignin pyrolysis are shown in Figure 2 and Figure 3.

For pure methane (Figure 2), equilibrium conversion increases with temperature, reaching 24.90 wt.% with an H_2_ selectivity of 66.65% at 1200 °C. The mass yields of solid carbon (C) and hydrogen (H_2_) at 1200 °C were 20.75 and 41.49 mol per kg of methane, respectively. The dominant reaction is methane pyrolysis (Equation (1)), which becomes thermodynamically favorable above approximately 650 °C. Concurrently, secondary reactions arise at elevated temperatures, such as Equation (16) (CH_4_ → C_2_H_4_ + H_2_), forming ethylene (C_2_H_4_). As the temperature further increases to 800 °C, the equilibrium methane conversion reaches approximately 2.41 wt%, at which point Equation (17) (2CH_4_ → C_2_H_2_ + 3H_2_) becomes detectable, resulting in acetylene (C_2_H_2_) formation. When the temperature exceeds 1000 °C, Equation (18) (C_2_H_2_ → 2C(s) + H_2_) intensifies, further promoting the generation of solid carbon (i.e., carbon deposition).

The main secondary reaction pathways at high temperatures include [20]:

(16) $$2{\mathrm{CH}}_{4}(g)⇌C_{2}H_{4}(g)+2H_{2}(g)\;(\mathrm{Ethylene}\;\mathrm{formation})$$

(17) $$2{\mathrm{CH}}_{4}(g)⇌C_{2}H_{2}(g)+3H_{2}(g)\;(\mathrm{Acetylene}\;\mathrm{formation})$$

(18) $$C_{2}H_{2}(g)⇌2C(s)+H_{2}(g)\;(\mathrm{Carbon}\;\mathrm{deposition})$$

While the complete mechanism of methane decomposition remains complex and is not entirely resolved, there is broad agreement that it proceeds via stepwise dehydrogenation [21]. The generally accepted pathway involves initial ethane (C_2_H_6_) formation, followed by its conversion to ethylene (C_2_H_4_) and finally to acetylene (C_2_H_2_) in the last decomposition stage [20]:

(19) $${2\mathrm{CH}}_{4}\overset{-H_{2}}{\to }C_{2}H_{6}\overset{-H_{2}}{\to }C_{2}H_{4}\overset{-H_{2}}{\to }C_{2}H_{2}\overset{-H_{2}}{\to }2C$$

Although high temperatures increase methane conversion from a thermodynamic standpoint, the substantial energy input required makes this strategy economically unattractive on an industrial scale. Simulated results for methane cracking are consistent with experimental data [22,23]. However, actual methane conversion at elevated temperatures remains low due to kinetic limitations: the uncatalyzed cracking rate is slow and the reaction strongly favors the formation of solid carbon, driven by its low Gibbs free energy. Consequently, achieving close to thermodynamic equilibrium in methane pyrolysis typically necessitates catalytic intervention to accelerate reaction kinetics [24,25].

For biomass components (Figure 3), the equilibrium product distribution is highly dependent on the feedstock type. At 1200 °C, lignin (LG) yields 33.65 mol/kg (40.38 wt%) of solid carbon, much higher than the 6.58 mol/kg (7.90 wt%) produced from cellulose (CE). In contrast, cellulose generates substantially more CO (30.3 mol/kg) compared to lignin (19.16 mol/kg). These results align closely with previous experimental findings [26,27]. The evolution of H_2_O, CH_4_, CO_2_, and H_2_ also demonstrates distinct temperature-dependent behaviors for each biomass type.

Solid carbon originates primarily from the decomposition of the raw biomass and decreases thermodynamically as the temperature rises, stabilizing at a residual level at higher temperatures [28]. Notably, in the 300–500 °C range, carbon depletion slows or even reverses slightly due to the Boudouard reaction (Equation (4)), where CO disproportions into solid carbon and CO_2_. At temperatures between 600 and 1200 °C, the water-gas reaction (Equation (8)) becomes predominant in consuming fixed carbon.

The evolution of water vapor during biomass thermal conversion exhibits three temperature-dependent stages, governed by material structure and thermodynamics [29]. Below 200 °C, moisture is released via physical desorption, involving primarily the liberation of free hydroxyl groups (-OH) without breaking any chemical bonds. Between 200 and 600 °C, chemical dehydration dominates through aliphatic and phenolic hydroxyl condensation and decarboxylation. Above 600 °C, water is consumed by gas-phase reactions like water–gas reaction (Equation (8), water-gas shift reaction (Equation (9)), and steam reforming (Equation (10)), leading to near-zero net production. The yield of water differs markedly between cellulose and lignin, reflecting their distinct compositions [27].

Both methane (CH_4_) and carbon dioxide (CO_2_) concentrations show similar trends with temperature, rising initially before declining toward zero. The corresponding peak production temperatures occur at approximately 300 °C for CH_4_ and 600 °C for CO_2_. The observed increase in methane can be attributed not only to direct biomass cracking but also to methanation reactions between C/CO and H_2_ (Equations (5)–(7), *T* < 600 °C). The rise in CO_2_ production likely originates from the Boudouard reaction (Equation (4), *T* < 600 °C) and water-gas shift reactions (Equation (9), *T* < 800 °C). At elevated temperatures (>800 °C), both CH_4_ and CO_2_ are consumed through methane pyrolysis (Equation (1)) and reforming reactions (Equations (10) and (11)), leading to their eventual depletion and concomitant production of CO and H_2_.

#### 3.2.2. Biomass–Methane Co-Pyrolysis

**Figure 4:**
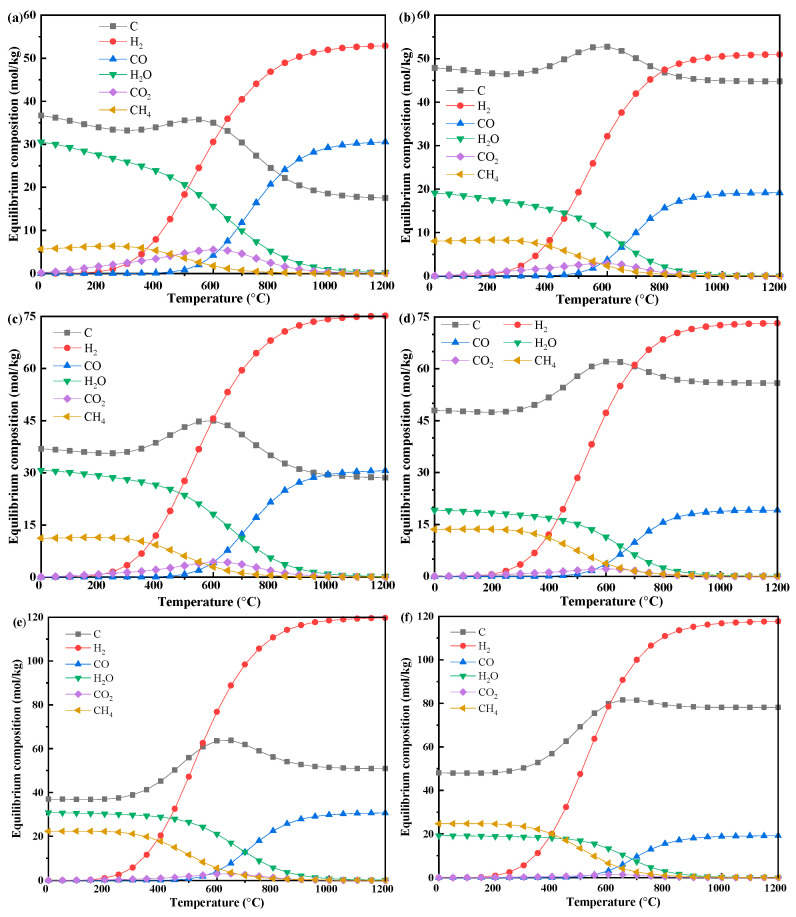
Influence of methane addition on the equilibrium compositions during biomass- methane co-pyrolysis under different temperatures (*p* = 1 bar, biomass 1 kg): (**a**) CE:CH_4_ = 4, (**b**) LG:CH_4_ = 4; (**c**) CE:CH_4_ = 2, (**d**) LG:CH_4_ = 2; (**e**) CE:CH_4_ = 1, (**f**) LG:CH_4_ = 1.

The equilibrium product distributions for biomass–methane mixtures at various mass ratios (ranging from 1:0.25 up to 1:1) are presented in Figure 4. The general temperature-dependent trends for major species (C, CH_4_, CO_2_, H_2_O, H_2_, CO) were consistent with those of pure biomass gasification, but the equilibrium concentrations were modified. Specifically, concentrations of C, CH_4_, and CO_2_ exhibit bell-shaped trends as temperature increases, while H_2_O content steadily declines. In contrast, H_2_ and CO show sigmoidal increases with temperature until reaching a plateau.

Importantly, higher methane content leads to a substantial enhancement in the yields of both solid carbon and H_2_, whereas the yield of CO remains remarkably stable across all conditions. At 1200 °C, for example, cellulose-based systems display an increase in carbon yield from 17.53 mol/kg to 50.86 mol/kg, and H_2_ yield rises from 52.89 mol/kg to 119.69 mol/kg as the biomass-to-methane ratio increases from 1:0.25 to 1:1, with CO maintaining approximately 30.6 mol/kg. Lignin exhibits similar temperature-dependent behavior but with characteristically higher carbon yields (44.77 to 78.16 mol/kg) and lower CO production (19.15 mol/kg) when compared to cellulose.

#### 3.2.3. H_2_/CO Ratio and Tar Formation Characteristics

**Figure 5:**
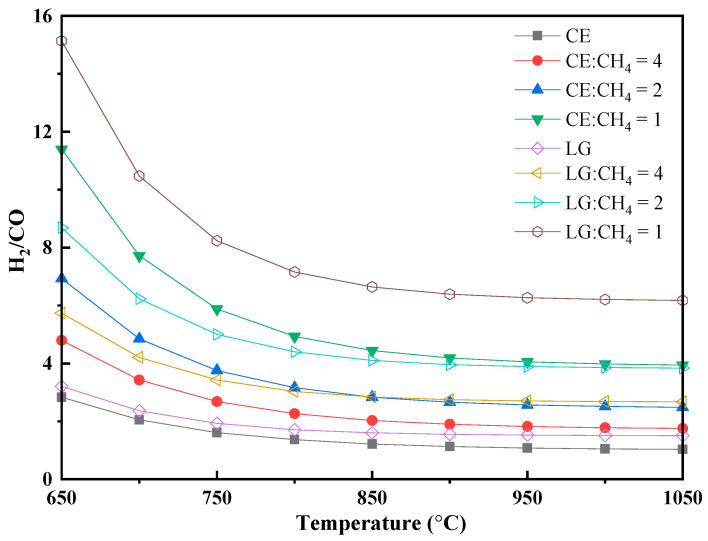
Change in the ratio of H2/CO for biomass–methane co-pyrolysis under different temperatures (*p* = 1 bar, biomass 1 kg).

The H_2_/CO ratio of the syngas is strongly influenced by both the biomass-to-methane ratio and the reaction temperature (Figure 5). Higher methane content and lower temperatures resulted in a higher H_2_/CO ratio. Temperature-dependent analysis reveals an inverse correlation between H_2_/CO ratio and reaction temperature above 700 °C. This behavior stems from competing reaction pathways: while methane pyrolysis (Equation (1)), water-gas (Equation (8)), and reforming reactions (steam reforming, Equation (10): CH_4_ + H_2_O → CO + 3H_2_; dry reforming, Equation (11): CH_4_ + CO_2_ → 2CO + 2H_2_) are all thermally activated, the reverse water-gas shift reaction (Equation (9), CO_2_ + H_2_ → CO + H_2_O) becomes increasingly dominant at higher temperatures, effectively consuming H_2_ and consequently reducing the H_2_/CO ratio in the product gas [30].

For pure biomass at 1200 °C, the syngas derived from lignin features a higher H_2_/CO ratio (1.50) than that from cellulose (1.01), reflecting lignin’s higher H_2_ yield and cellulose’s greater CO production per mass. When the biomass-to-methane ratio is set at 1:1, the H_2_/CO ratios increase dramatically to 3.91 for cellulose and 6.15 for lignin systems. These elevated H_2_/CO ratios (>2) are considered optimal for downstream applications such as liquid fuel synthesis via Fischer–Tropsch processes [8,10].

**Figure 6:**
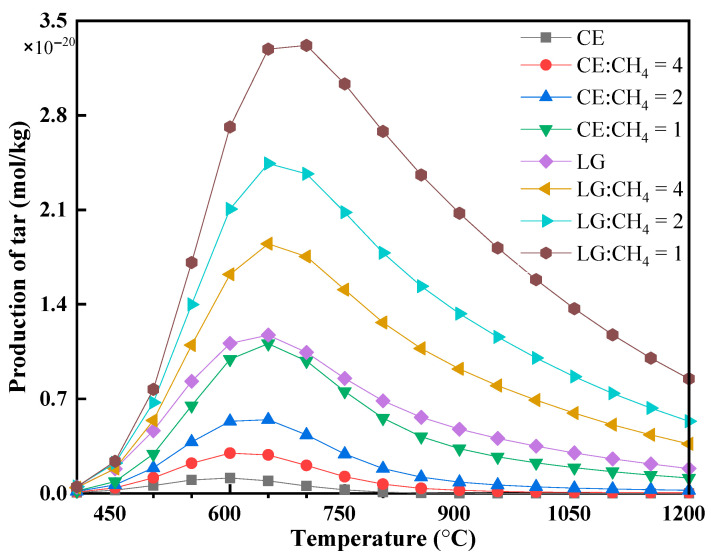
Tar formation during biomass-methane co-pyrolysis under different temperatures (*p* = 1 bar, biomass 1 kg).

Understanding tar formation and its decomposition is equally important for effective gasification process design and catalyst optimization [8,31]. The equilibrium yield of tar exhibits a unimodal trend with temperature—increasing to a peak near 600 °C before declining at higher temperatures (Figure 6). Across the temperature spectrum, lignin consistently produces more tar than cellulose. Additionally, supplementing the biomass with methane leads to substantially higher equilibrium tar yields compared to pure biomass gasification under equivalent conditions.

### 3.3. Energy Recovery Efficiency

The energy of products and corresponding energy recovery efficiency were quantitatively assessed using the calculation methods defined in Equations (13)–(15), with detailed results presented in Table 2.

For pure feedstocks, energy recovery efficiency improves as reaction temperature increases. In pure methane pyrolysis, the energy content of the gas products rises from 36.43 MJ/kg CH_4_ at 800 °C to 38.53 MJ/kg CH_4_ at 1000 °C. Correspondingly, the energy recovery efficiency improves from 72.68% to 76.87%. However, the incremental energy gain of just 2.1 MJ/kg CH_4_ over this temperature range is economically disadvantageous given the significant energy input necessary for operating at higher temperatures.

For pure biomass pyrolysis, both cellulose and lignin show increased energy output with rising temperature, though their energy recovery efficiencies differ notably. Cellulose undergoes a particularly dramatic improvement: product energy increases from 13.4 MJ/kg biomass (*E*_η_ = 82.74%) at 800 °C to 16.1 MJ/kg biomass (*E*_η_ = 99.36%) at 1000 °C, reflecting near-complete conversion at the highest temperature assessed. By contrast, lignin’s efficiency remains substantially lower, reaching only 15.13 MJ/kg (61.77%) at 800 °C and 16.08 MJ/kg (65.62%) at 1000 °C.

In the biomass–methane co-pyrolysis system, increasing methane loading (from 0 to 1 kg CH_4_/kg biomass) boosts total energy output, but its effect on energy recovery efficiency varies by biomass type. For cellulose, *E*_η_ d decreases with methane addition—from 82.74% (pure biomass, 800 °C) to 60.74% at the same temperature with methane—while for lignin, *E*_η_ increases from 61.77% to 68.34% at 800 °C as methane content rises.

## 4. Discussion

### 4.1. Thermodynamic Driving Forces and Reaction Pathways

The calculated ΔG serves as a fundamental indicator of reaction spontaneity and equilibrium in biomass–methane co-pyrolysis systems. A critical thermodynamic threshold is observed at 700 °C: below this temperature, competing methanation and carbon deposition reactions are favored, consuming H_2_ and depositing solid carbon, which hinders efficient syngas production [19]. Above 700 °C, the equilibrium shifts toward methane cracking and steam or dry reforming reactions, resulting in a substantial increase in both total syngas yield and the H_2_/CO ratio, making the syngas especially suitable for downstream applications such as Fischer–Tropsch synthesis (H_2_/CO > 2) [32,33]. Additionally, targeted steam injection can further suppress undesirable side reactions such as methanation and carbon deposition, allowing selective production of high-purity, hydrogen-rich syngas [19]. These thermodynamic findings align closely with the experimental results of Lalsare et al. [7], underscoring that, upon methane addition, the shift in product distribution is driven not by methane accumulation, but by its active participation in reforming reactions that enhance hydrogen yield. Therefore, maintaining reaction conditions above 700 °C with controlled steam addition is key to maximizing H_2_-rich syngas while minimizing byproduct formation during biomass–methane co-pyrolysis.

### 4.2. Influence of Biomass Molecular Structure

The pronounced differences in product distributions between cellulose and lignin (Figure 3) arise from their distinct molecular architectures, which dictate unique thermal decomposition pathways and resultant equilibrium states [27]. These structural contrasts manifest primarily in two ways: the stability of the carbon framework and the characteristics of oxygen-containing functional groups.

Firstly, lignin features a highly cross-linked aromatic network stabilized by strong C–C and ether bonds (>300 kJ/mol), imparting high thermal stability that encourages carbon retention and condensation reactions—and consequently, higher solid carbon and tar yields [28]. In contrast, cellulose possesses a linear polysaccharide structure with weaker glycosidic bonds (70 kJ/mol), facilitating depolymerization and volatilization and resulting in higher gas yields.

Secondly, functional group composition impacts gas-phase product speciation. Lignin contains methoxy (-OCH_3_) and alkyl side chains that act as methyl donors, leading to significantly increased methane production via methyl radical recombination [31]. Cellulose, which lacks these groups, produces minimal methane. Its high concentration of labile carboxyl (-COOH) groups (*E*_a_ ≈ 90 kJ/mol) makes it a major source of CO_2_ through decarboxylation [34], while CO_2_ from lignin typically forms via rearrangement of more stable carbonyl groups.

Further distinctions arise in the formation of CO and H_2_. Cellulose efficiently generates CO through β-scission of carbohydrate rings and benefits from higher oxygen mobility, yielding 1.58 times more CO than lignin at equilibrium. Cellulose’s aliphatic chain structure also more efficiently undergoes aromatization and dehydrogenation of furanic intermediates at high temperature (>600 °C), promoting slightly higher H_2_ yields than lignin [28,29]. These observations highlight cellulose’s greater facility for converting oxygen functional groups to CO and its molecular structure to H_2_.

In summary, the observed product distribution is fundamentally governed by the molecular architecture of the biomass: lignin’s stable aromatic and cross-linked framework favors carbon and methane formation, while cellulose’s reactive aliphatic polysaccharide structure favors gasification and efficient syngas production.

### 4.3. Synergistic Mechanisms in Co-Pyrolysis

The analysis of product evolution across temperatures reveals distinct regimes governed by different reaction pathways. At low temperatures (<600 °C), methane remains mostly inert, and any CH_4_ detected below 300 °C arises from biomass decomposition, serving as a baseline for methane’s influence. As temperature increases above 800 °C, reforming reactions begin to dominate, converting primary pyrolysis products into solid carbon (C), CO, and H_2_, establishing unique high-temperature equilibrium states. Notably, for both cellulose and lignin, increasing the methane input consistently yields higher amounts of reduced products (C, CH_4_, H_2_) and inhibits oxidized species (CO_2_, CO), indicating a reproducible shift toward more reductive product composition [7,13].

At high temperatures (800–1200 °C), the impact of methane addition becomes pronounced. Thermodynamic decoupling analysis—subtracting pure component pyrolysis yields from the co-pyrolysis output—demonstrates clear synergistic effects:

(1) Methane enhances biomass conversion: At a 1:1 ratio, cellulose produces an additional 30.10 mol C/kg and 78.20 mol H_2_/kg, while lignin yields an extra 57.41 mol C/kg and 76.20 mol H_2_/kg compared to theoretical values from individual pyrolysis.
(2) Biomass catalyzes methane cracking: Co-pyrolysis significantly boosts methane conversion, raising it from 24.90% for pure methane to over 53% with biomass present, nearly doubling the yield while maintaining high H_2_ selectivity (~67%).

This mutual enhancement demonstrates a profound thermodynamic and kinetic synergy. The observed enhancement in H_2_ yield and the shift toward a more reductive environment suggest a potential mechanism involving reactive intermediates. Consistent with previous kinetic research, methane pyrolysis generates radicals (such as ·H and ·CH_3_) that promote hydrodeoxygenation (HDO) and cracking during biomass conversion [7]. Therefore, the thermodynamic shift observed in our model can be rationally interpreted as a consequence of such radical chain interactions. Simultaneously, the generated biochar functions catalytically, providing active surfaces for these radical processes and facilitating carbon nucleation, thus lowering the activation energy for methane cracking [7,35]. The overall shift toward a reductive environment is fundamentally driven by the high H/C_eff_ ratio of methane (4.0), which counterbalances the comparatively low H/C_eff_ ratio of biomass (0.1–1.5).

In essence, co-pyrolysis synergy improves product quality via two primary mechanisms: radical-mediated tar cracking, which increases H_2_ yield, and optimization of the overall H/C ratio for producing desirable syngas [7,13,16]. Furthermore, the methane decomposition pathway (CH_4_ → C + 2H_2_) offers a thermodynamically feasible route with near-zero CO_2_ emissions, effectively complementing the carbon-neutral nature of biomass and aligning with the development of low-carbon energy technologies [3].

### 4.4. Implications for Process Optimization and Challenges

The H_2_/CO ratio serves as a critical indicator for evaluating syngas quality and determining its suitability for downstream applications, particularly catalytic conversion processes. The inverse correlation between H_2_/CO ratio and temperature above 700 °C is due to the increasing dominance of the reverse water-gas shift reaction (RWGS), which preferentially consumes H_2_ at higher temperatures [30]. The ability to tune the H_2_/CO ratio from 1.0 to over 6.0 by adjusting the biomass-to-methane ratio and temperature (Figure 5) provides crucial flexibility for tailoring syngas to specific downstream applications, such as Fischer–Tropsch synthesis [8].

However, pursuing an optimal H_2_/CO ratio must be balanced against tar formation challenges. Tar yield follows a unimodal trend with temperature, peaking around 600 °C. Notably, lignin produces significantly more tar than cellulose due to its native cross-linked aromatic structure, which serves as a stable precursor for condensation [27]. Methane addition, especially at higher ratios (up to 1 kg CH_4_ per kg biomass), further elevates equilibrium tar yield. Early tar formation is dominated by devolatilization, generating light aromatics (benzene, toluene) and phenolics [30]. Above 700 °C, continued heating leads to secondary cracking and fusion into polycyclic aromatic hydrocarbons (PAHs)-exemplified by biphenyl (C_12_H_10_) and naphthalene (C_10_H_8_) formation (Equation (20)) [36]. These heavier tars can eventually be cracked into syngas and solid carbon. Methane may inhibit tar decomposition (driving formation of heavier compounds via methyl radical insertion) [36], which aligns with observed increases in tar yield.

(20) $${2C}_{6}H_{6}\overset{-H_{2}}{\to }C_{12}H_{10}\left(\mathrm{Biphenyl}\right)\overset{-H_{2}}{\to }C_{10}H_{8}\left(\mathrm{Naphthalene}\right)$$

Despite methane’s substantial boost to net H_2_ production (by 40–160% over theoretical methane-only values), the tradeoff is increased tar—a precursor for carbon deposition and reactor fouling. Striking a balance is essential. Thermodynamic analysis identifies a 50% methane blending ratio as optimal: it maximizes H_2_ yield while limiting carbon formation. This optimum is corroborated by experimental findings (e.g., Korpeh et al. [1]), who reported enhanced combustion properties at the same proportion. Such modeling–experimental agreement underscores a robust synergistic effect at this ratio, achieving both thermodynamic and operational benefits.

Additionally, the process yields significant solid carbon (up to 78.16 mol/kg for lignin) alongside hydrogen-rich syngas. This solid product is biochar—a porous, functionalized carbon material [21,37]. Unlike undesirable amorphous coke, biochar is a valuable co-product for use as adsorbent, catalyst support, or soil amendment [37,38]. Its high yield not only avoids operational issues but also presents opportunities for circular resource utilization, supporting process economics and sustainability.

### 4.5. Energetic Performance and Feedstock Selection

Analysis of energy recovery efficiency (*E*_η_) provides critical insights for practical process optimization. Cellulose achieves near-complete conversion (*E*_η_ = 99.36% at 1000 °C), while lignin, owing to its recalcitrant aromatic structure, achieves significantly lower efficiency (65.62% at the same temperature) [27]. Methane addition further highlights these differences: for cellulose, co-processing increases total energy output but reduces overall system efficiency due to methane’s moderate conversion rate. By contrast, co-pyrolysis with lignin is highly advantageous, simultaneously boosting both total energy and process efficiency by compensating for lignin’s lower inherent conversion.

Furthermore, the modest energy gain (2.1 MJ/kg) associated with a 200 °C temperature increase in pure methane pyrolysis, compared to the required energy input, highlights the economic obstacles of uncatalyzed thermal decomposition—a limitation rooted in thermodynamic equilibrium [39,40].

Collectively, these findings inform the design of industrial co-pyrolysis processes, suggesting the strategic pairing of refractory biomass streams (such as lignin) with methane-rich gas sources to maximize energy recovery and conversion efficiency, thereby enhancing economic viability.

## 5. Conclusions

This study establishes a comprehensive thermodynamic and mechanistic framework for biomass-methane co-pyrolysis through Gibbs free energy minimization modeling. The key findings reveal that the process is governed by a critical temperature threshold of 700 °C, below which methanation and carbon deposition prevail, and above which cracking and reforming reactions dominate to produce high-purity syngas.

The introduction of methane induces a fundamental shift in reaction pathways toward reduction, significantly enhancing solid carbon and hydrogen yields while suppressing oxidized products (CO/CO_2_). This synergistic effect is bidirectional: methane-derived reactive intermediates (e.g., ·H and ·CH_3_) promote the hydrodeoxygenation and stabilization of biomass-derived carbon, while the in situ formed biochar acts as an effective catalyst, boosting methane conversion from 25% to over 53% while maintaining high hydrogen selectivity (~67%).

The distinct molecular structures of biomass components critically influence the outcomes. Lignin’s aromatic network favors carbon retention and tar formation, whereas cellulose’s aliphatic chains lead to higher CO yields. Consequently, the synergistic enhancement in energy efficiency is more pronounced for recalcitrant lignin than for highly convertible cellulose.

Optimization analysis identifies an optimal methane-to-biomass ratio of 50% and a temperature of 1000 °C, which collectively maximize hydrogen yield, limit carbon deposition and tar formation, and yield a tunable H_2_/CO ratio (1.0–6.0) suitable for downstream applications like Fischer–Tropsch synthesis.

In summary, these insights provide a fundamental thermodynamic foundation for designing efficient co-pyrolysis processes. The work highlights the potential of biomass-methane co-processing as a versatile strategy for the sustainable co-production of high-quality syngas and carbon materials with minimized undesirable byproducts.

## Figures and Tables

**Table 1:** Thermodynamic parameters of substances at 25 °C.

	Formula	MW (g/mol)	Δ*H*_f_° (kJ/mol)	Δ*S*_f_° (J/K/mol)	HHV (kJ/mol)	LHV (kJ/mol)	Heat of Combustion (MJ/kg)
Methane	CH_4_	16	−74.81	186.3	−891.0	−802.0	−56.0
Cellulose	C_6_H_10_O_5_	162	−1019.0	181.0	−2624.4	−	−16.2
Lignin	C_11_H_12_O_4_	208	−729.31	239.8	−5096	−	−24.5
Oxygen	O_2_	32	0	205.2			
Water (liquid)	H_2_O (l)	18	−285.83	69.9			
Water (gas)	H_2_O (g)	18	−241.80	188.8			
Carbon dioxide	CO_2_	44	−393.51	213.8			
Carbon monoxide	CO	28	−110.53	197.7	−284.0	−283.0	−10.0
Carbon	C	12	0	5.7	−394.0	−110.5	−33.0
Hydrogen	H_2_	2	0	130.7	−286.0	−244.0	−143.0
Benzene	C_6_H_6_	78	−82.93	269.31	−3267.6	−3129.6	−41.8

Note: MW stands for molecular weight, g/mol; Δ*H*_f_° represents standard enthalpy of formation, kJ/mol; Δ*S*_f_° represents standard entropy of formation, J/K/mol; HHV stands for higher heating value, kJ/mol; LHV stands for lower heating value, kJ/mol.

**Table 2:** Energy of products and energy recovery efficiency of co-pyrolysis of biomass and methane under different temperatures.

Biomass-to-Methane Ratio			*E*_out_ (MJ)			*E*_η_ (%)		
CE ^a^	LG ^a^	CH_4_^b^	800 °C	900 °C	1000 °C	800 °C	900 °C	1000 °C
		1	36.43	37.17	38.53	72.68	74.16	76.87
1			13.40	15.14	16.10	82.74	93.45	99.36
1		0.25	20.20	22.14	23.03	70.33	77.07	80.14
1		0.5	26.94	28.95	29.78	65.30	70.16	72.16
1		1	40.28	42.38	43.16	60.74	63.89	65.08
	1		15.13	15.86	16.08	61.77	64.71	65.62
	1	0.25	23.63	24.25	24.54	63.81	65.49	66.28
	1	0.5	32.91	33.59	33.78	66.41	67.78	68.16
	1	1	51.00	52.42	52.91	68.34	70.25	70.90

^a^ denotes biomass as the reaction feedstock, with input mass in kg; ^b^ denotes methane as the reaction feedstock, with input mass in kg; CE stands for cellulose; LG stands for lignin; *E*_out_ represents output energy, MJ; *E*_η_ represents energy recovery efficiency, %. The biomass-to-methane input ratio is expressed on a mass basis, with biomass fixed at 1 kg. The mass of methane required for co-processing is calculated based on the specified mass ratio.

## Data Availability

The original contributions presented in this study are included in the article. Further inquiries can be directed to the corresponding author(s).

## Abbreviations

The following abbreviations are used in this manuscript:

CE	Cellulose
CGE	Cold gas efficiency
G	Guaiacyl lignin subunit
H	*p*-hydroxyphenyl
HDO	Hydrodeoxygenation
HHV	Higher heating value
LHV	Lower heating value
LG	Lignin (Used as a label in figures and tables)
MJ	Megajoule
PAHs	Polycyclic aromatic hydrocarbons
S	Syringyl lignin subunit
WGSR	Water–gas shift reaction
